# Etoricoxibium picrate

**DOI:** 10.1107/S1600536810050993

**Published:** 2010-12-11

**Authors:** Jerry P. Jasinski, Ray J. Butcher, M. S. Siddegowda, H. S. Yathirajan, A. R. Ramesha

**Affiliations:** a Department of Chemistry, Keene State College, 229 Main Street, Keene, NH 03435-2001, USA; b Department of Chemistry, Howard University, 525 College Street NW, Washington, DC 20059, USA; c Department of Studies in Chemistry, University of Mysore, Manasagangotri, Mysore 570 006, India; d RL Fine Chem., Bangalore 560 064, India, Department of Studies in Chemistry, Mangalore University, Mangalagangotri 574 199, India

## Abstract

In the cation of the title salt (systematic name: 5-{5-chloro-3-[4-(methyl­sulfon­yl)phen­yl]-2-pyrid­yl}-2-methyl­pyridinium 2,4,6-trinitro­phenolate), C_18_H_16_ClN_2_O_2_S^+^·C_6_H_2_N_3_O_7_^−^, the mean planes of the two pyridine rings in the bipyridine unit are twisted by 33.9 (2)° with respect to each other. The dihedral angles between the mean planes of the sulfonyl­benzene ring and the chloro­pyridine and methyl­pyridine rings are 51.2 (0) and 49.3 (9)°, respectively. The picrate anion inter­acts with the protonated N atom through a bifurcated N—H⋯(O,O) hydrogen bond, forming an *R*_1_^2^(6) ring motif with the N atom from the methyl­pyridine group of an adjacent cation. N—H⋯O hydrogen bonds, weak C—H⋯O and π–π stacking inter­actions [centroid–centroid distances = 3.8192 (9)and 3.6749 (9)] occur in the crystal packing, creating a two-dimensional network structure along [110].

## Related literature

For the selective COX-2 inhibitor etoricoxib, see: Patrignani *et al.* (2003 ▶). For background to coxibs, traditional non-steroidal anti-inflammatory drugs, see: Rimon *et al.* (2010 ▶); Shriner *et al.* (1980) ▶; Patrignani *et al.* (2003 ▶). For related structures, see: Malathy Sony *et al.* (2005 ▶); Vasu Dev *et al.* (1999 ▶); Yathirajan *et al.* (2005 ▶). For standard bond lengths, see: Allen *et al.* (1987 ▶). 
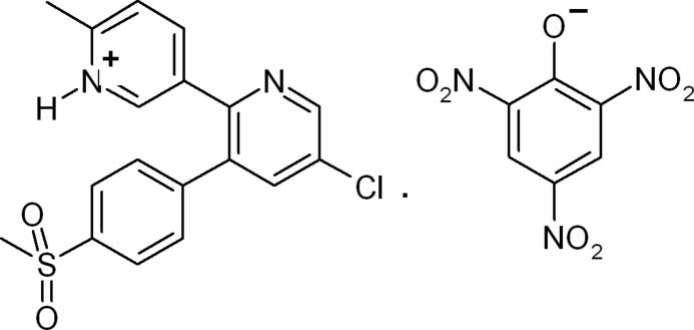


## Experimental

### 

#### Crystal data

C_18_H_16_ClN_2_O_2_S^+^·C_6_H_2_N_3_O_7_^−^
*M* *_r_* = 587.94
Monoclinic, 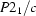

*a* = 9.0250 (1) Å
*b* = 12.7496 (1) Å
*c* = 21.8011 (3) Å
β = 98.114 (1)°
*V* = 2483.43 (5) Å^3^
*Z* = 4
Cu *K*α radiation
μ = 2.74 mm^−1^
*T* = 123 K
0.48 × 0.42 × 0.24 mm

#### Data collection

Oxford Diffraction Xcalibur Ruby Gemini diffractometer
Absorption correction: multi-scan (*CrysAlis RED*; Oxford Diffraction, 2007 ▶) *T*_min_ = 0.607, *T*_max_ = 1.000
9467 measured reflections
4932 independent reflections
4454 reflections with *I* > 2σ(*I*)
*R*_int_ = 0.021

#### Refinement

*R*[*F* ^2^ > 2σ(*F* ^2^)] = 0.038
*wR*(*F* ^2^) = 0.104
*S* = 1.03
4932 reflections
363 parameters
H-atom parameters constrained
Δρ_max_ = 0.44 e Å^−3^
Δρ_min_ = −0.38 e Å^−3^

### 

Data collection: *CrysAlis PRO* (Oxford Diffraction, 2007 ▶); cell refinement: *CrysAlis PRO*; data reduction: *CrysAlis RED* (Oxford Diffraction, 2007 ▶); program(s) used to solve structure: *SHELXS97* (Sheldrick, 2008 ▶); program(s) used to refine structure: *SHELXL97* (Sheldrick, 2008 ▶); molecular graphics: *SHELXTL* (Sheldrick, 2008 ▶); software used to prepare material for publication: *SHELXTL*.

## Supplementary Material

Crystal structure: contains datablocks global, I. DOI: 10.1107/S1600536810050993/zl2336sup1.cif

Structure factors: contains datablocks I. DOI: 10.1107/S1600536810050993/zl2336Isup2.hkl

Additional supplementary materials:  crystallographic information; 3D view; checkCIF report

## Figures and Tables

**Table 1:** Hydrogen-bond geometry (Å, °)

*D*—H⋯*A*	*D*—H	H⋯*A*	*D*⋯*A*	*D*—H⋯*A*
N2*A*—H2*AB*⋯O1*B*	0.88	1.79	2.6588 (18)	172
N2*A*—H2*AB*⋯O7*B*	0.88	2.46	2.8898 (19)	111
C2*A*—H2*AA*⋯O1*A*^i^	0.95	2.56	3.455 (2)	156
C9*A*—H9*AA*⋯O1*B*	0.98	2.60	3.357 (2)	134
C13*A*—H13*A*⋯O2*A*^ii^	0.95	2.35	3.294 (2)	173
C18*A*—H18*C*⋯O2*B*^iii^	0.98	2.38	3.249 (2)	147
C5*A*—H5*AA*⋯O6*B*^iv^	0.95	2.45	3.329 (2)	153
C7*A*—H7*AA*⋯O4*B*^v^	0.95	2.52	3.326 (2)	143

Symmetry codes: (i) 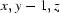; (ii) 
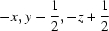
; (iii) 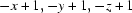; (iv) 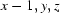; (v) 
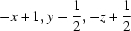
.

**Fig. 1.**
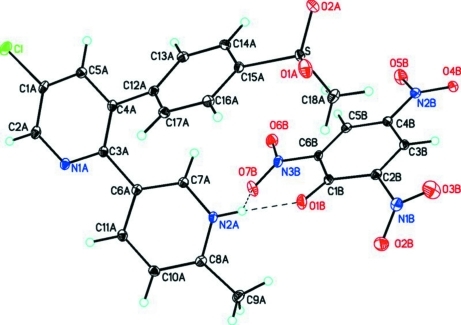
Molecular structure of the title compound showing the atom labeling scheme and 50% probability displacement ellipsoids. Dashed lines indicate N—H···O hydrogen bonds between the cation and anion and a R12(6) ring motif.

**Fig. 2.**
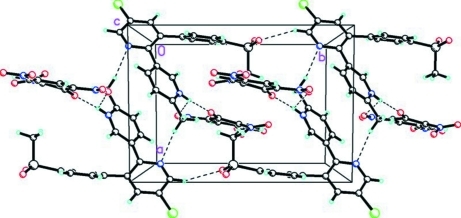
Packing diagram of the title compound viewed down the c axis. Dashed lines indicate N—H···O hydrogen bonds and weak C—H···O intermolecular interactions creating a 2-D network along the 110.

## supplementary crystallographic information

### Comment

Coxibs are the traditional non-steroidal anti-inflammatory drugs that counter the positive effects of aspirin in preventing blood clots. The research, published in the Proceedings of the National Academy of Sciences (Rimon *et al.*, 2010)), indicates that people who are taking aspirin and coxibs together are in fact inhibiting the aspirin's effectiveness in preventing heart attacks and strokes. Some of the important class of coxib drugs are valdecoxib, celecoxib, rofecoxib, lumiracoxib, etoricoxib *etc*. Etoricoxib (brand name Arcoxia worldwide; also Algix and Tauxib in Italy) is a novel selective COX-2 inhibitor (Patrignani *et al.*, 2003). Like any other COX-2 selective inhibitor, etoricoxib selectively inhibits isoform 2 of the enzyme *cyclo*-oxigenase (COX-2). The crystal structure of valdecoxib, a non-steroidal anti-inflammatory drug (Malathy Sony *et al.*, 2005), a pseudopolymorph of valdecoxib (Yathirajan *et al.*, 2005) and celecoxib, a COX-II inhibitor (Vasu Dev *et al.*, 1999) have been reported.  In the view of the importance of etoricoxib, this paper presents the crystal structure of the title compound, etoricoxib picrate.

In the crystal structure of the title compound, C_18_H_16_ClN_2_O_2_S^+^. C_6_H_2_N_3_O_7_^-^, there is one cation-anion pair in the asymmetric unit (Fig. 1). In the cation, the mean planes of the two pyridine rings in the bipyridine moiety are twisted by 33.9 (2)° against each other. The dihedral angle between the mean planes of the sulfonylbenzene ring and the chloropyridine and methylpyridine rings are 51.2 (0)° and 49.3 (9)°, respectively. The picrate anion interacts with the protonated N atom through a bifurcated N—H···O hydrogen bond forming a *R*_1_^2^(6) ring motif with the N atom from the methylpyridine group of an adjacent cation.

The dihedral angles between the mean planes of the anion benzene ring and three chloropyridine, methylpyridine and sulfonylbenzene rings of the cation are 53.9 (1)°, 49.3 (9)° and 3.8 (8)°, respectively. The mean planes of the two *o*-NO_2_ and single *p*-NO_2_ groups in the picrate anion are twisted by 3.0 (5)°, 30.4 (7)° and 6.5 (9)° with respect to the mean plane of the 6-membered benzene ring. Bond distances and angles are in normal ranges (Allen *et al.*, 1987). N—H···O hydrogen bonds, weak C—H···O (Table 1) and π–π stacking interactions (Table 2) dominate the crystal packing creating an infinite 2-D network structure along the 110 (Fig. 2).

### Experimental

Etoricoxib (3.59 g, 0.01 mmol) and picric acid (2.29 g, 0.01 mmol) in the ratio 1:1 were mixed together in a hot methanol solution. The mixture was warmed to 330 K for few minutes. The resultant precipitate was dried and recrystallized using DMSO. Crystals of the title compound were obtained by the slow evaporation of DMSO solution at room temperature after a few days. (m.p.: 463 – 465 K).

### Refinement

All of the H atoms were placed in their calculated positions and then refined using the riding model with Atom—H lengths of 0.95Å (CH), 0.98Å (CH_3_) or 0.88Å (NH). Isotropic displacement parameters for these atoms were set to 1.18 times (NH), 1.18–1.22 (CH) or 1.50–1.51 (CH_3_) times *U*_eq_ of the parent atom.

### Crystal data

**Table:**

C_18_H_16_ClN_2_O_2_S^+^·C_6_H_2_N_3_O_7_^−^	*F*(000) = 1208
*M**_r_* = 587.94	*D*_x_ = 1.573 Mg m^−^^3^
Monoclinic, *P*2_1_/*c*	Cu *K*α radiation, λ = 1.54178 Å
Hall symbol: -P 2ybc	Cell parameters from 6755 reflections
*a* = 9.0250 (1) Å	θ = 5.0–74.0°
*b* = 12.7496 (1) Å	µ = 2.74 mm^−^^1^
*c* = 21.8011 (3) Å	*T* = 123 K
β = 98.114 (1)°	Prism, pale yellow
*V* = 2483.43 (5) Å^3^	0.48 × 0.42 × 0.24 mm
*Z* = 4	

### Data collection

**Table:**

Oxford Diffraction Xcalibur Ruby Gemini diffractometer	4932 independent reflections
Radiation source: Enhance (Cu) X-ray Source	4454 reflections with *I* > 2σ(*I*)
graphite	*R*_int_ = 0.021
Detector resolution: 10.5081 pixels mm^-1^	θ_max_ = 74.2°, θ_min_ = 5.0°
ω scans	*h* = −11→9
Absorption correction: multi-scan (*CrysAlis RED*; Oxford Diffraction, 2007)	*k* = −15→15
*T*_min_ = 0.607, *T*_max_ = 1.000	*l* = −26→18
9467 measured reflections	

### Refinement

**Table:**

Refinement on *F*^2^	Primary atom site location: structure-invariant direct methods
Least-squares matrix: full	Secondary atom site location: difference Fourier map
*R*[*F*^2^ > 2σ(*F*^2^)] = 0.038	Hydrogen site location: inferred from neighbouring sites
*wR*(*F*^2^) = 0.104	H-atom parameters constrained
*S* = 1.03	*w* = 1/[σ^2^(*F*_o_^2^) + (0.0651*P*)^2^ + 1.3666*P*] where *P* = (*F*_o_^2^ + 2*F*_c_^2^)/3
4932 reflections	(Δ/σ)_max_ = 0.001
363 parameters	Δρ_max_ = 0.44 e Å^−^^3^
0 restraints	Δρ_min_ = −0.38 e Å^−^^3^

### Special details

**Table:**

Geometry. All e.s.d.'s (except the e.s.d. in the dihedral angle between two l.s. planes) are estimated using the full covariance matrix. The cell e.s.d.'s are taken into account individually in the estimation of e.s.d.'s in distances, angles and torsion angles; correlations between e.s.d.'s in cell parameters are only used when they are defined by crystal symmetry. An approximate (isotropic) treatment of cell e.s.d.'s is used for estimating e.s.d.'s involving l.s. planes.
Refinement. Refinement of *F*^2^ against ALL reflections. The weighted *R*-factor *wR* and goodness of fit *S* are based on *F*^2^, conventional *R*-factors *R* are based on *F*, with *F* set to zero for negative *F*^2^. The threshold expression of *F*^2^ > σ(*F*^2^) is used only for calculating *R*-factors(gt) *etc*. and is not relevant to the choice of reflections for refinement. *R*-factors based on *F*^2^ are statistically about twice as large as those based on *F*, and *R*- factors based on ALL data will be even larger.

### Fractional atomic coordinates and isotropic or equivalent isotropic displacement parameters (Å^2^)

**Table:**

	*x*	*y*	*z*	*U*_iso_*/*U*_eq_	
Cl	−0.27058 (5)	−0.16223 (3)	0.30221 (2)	0.02597 (12)	
S	0.06564 (4)	0.53861 (3)	0.361812 (19)	0.01753 (11)	
O1A	0.02079 (16)	0.59365 (10)	0.41361 (7)	0.0288 (3)	
O2A	0.00758 (15)	0.57350 (10)	0.30009 (6)	0.0287 (3)	
N1A	0.06432 (15)	−0.08812 (11)	0.43234 (6)	0.0165 (3)	
N2A	0.42035 (14)	0.18006 (10)	0.47118 (6)	0.0144 (3)	
H2AB	0.4697	0.2297	0.4547	0.017*	
C1A	−0.13587 (17)	−0.09020 (13)	0.34908 (8)	0.0169 (3)	
C2A	−0.04308 (18)	−0.14008 (13)	0.39641 (8)	0.0188 (3)	
H2AA	−0.0564	−0.2128	0.4034	0.023*	
C3A	0.08116 (17)	0.01546 (12)	0.42314 (7)	0.0137 (3)	
C4A	−0.01720 (17)	0.07282 (13)	0.37997 (7)	0.0141 (3)	
C5A	−0.12491 (17)	0.01675 (13)	0.34070 (7)	0.0160 (3)	
H5AA	−0.1893	0.0515	0.3089	0.019*	
C6A	0.21601 (17)	0.06182 (12)	0.46066 (7)	0.0137 (3)	
C7A	0.29848 (17)	0.13998 (12)	0.43700 (7)	0.0144 (3)	
H7AA	0.2683	0.1654	0.3962	0.017*	
C8A	0.47188 (17)	0.14885 (13)	0.52934 (7)	0.0150 (3)	
C9A	0.60555 (19)	0.20386 (14)	0.56235 (9)	0.0224 (4)	
H9AA	0.6164	0.2722	0.5429	0.034*	
H9AB	0.6952	0.1615	0.5600	0.034*	
H9AC	0.5930	0.2140	0.6059	0.034*	
C10A	0.39764 (18)	0.06695 (13)	0.55375 (8)	0.0164 (3)	
H10A	0.4335	0.0405	0.5938	0.020*	
C11A	0.27134 (18)	0.02370 (13)	0.51971 (8)	0.0156 (3)	
H11A	0.2214	−0.0327	0.5366	0.019*	
C12A	−0.01200 (16)	0.18932 (13)	0.37508 (7)	0.0142 (3)	
C13A	−0.00357 (18)	0.23552 (13)	0.31785 (8)	0.0161 (3)	
H13A	−0.0098	0.1934	0.2816	0.019*	
C14A	0.01398 (18)	0.34369 (13)	0.31387 (7)	0.0160 (3)	
H14A	0.0206	0.3759	0.2751	0.019*	
C15A	0.02176 (17)	0.40413 (12)	0.36752 (8)	0.0146 (3)	
C16A	0.00510 (18)	0.36004 (13)	0.42436 (7)	0.0156 (3)	
H16A	0.0063	0.4028	0.4601	0.019*	
C17A	−0.01336 (18)	0.25191 (13)	0.42787 (7)	0.0159 (3)	
H17A	−0.0269	0.2204	0.4662	0.019*	
C18A	0.2626 (2)	0.53815 (15)	0.36775 (9)	0.0259 (4)	
H18A	0.2983	0.6096	0.3621	0.039*	
H18B	0.2933	0.4923	0.3357	0.039*	
H18C	0.3056	0.5123	0.4088	0.039*	
O1B	0.56283 (15)	0.34250 (10)	0.43011 (6)	0.0259 (3)	
O2B	0.72286 (18)	0.50574 (12)	0.48473 (6)	0.0361 (4)	
O3B	0.6532 (2)	0.64555 (11)	0.43287 (7)	0.0396 (4)	
O4B	0.71450 (14)	0.62738 (10)	0.21604 (6)	0.0235 (3)	
O5B	0.69640 (18)	0.47710 (12)	0.16915 (6)	0.0342 (3)	
O6B	0.60280 (16)	0.16893 (10)	0.27056 (6)	0.0274 (3)	
O7B	0.58882 (16)	0.16083 (10)	0.36843 (6)	0.0282 (3)	
N1B	0.67468 (17)	0.55080 (12)	0.43669 (7)	0.0214 (3)	
N2B	0.69422 (16)	0.53193 (12)	0.21568 (7)	0.0198 (3)	
N3B	0.59954 (15)	0.21143 (11)	0.32115 (7)	0.0192 (3)	
C1B	0.60694 (17)	0.37936 (13)	0.38317 (8)	0.0162 (3)	
C2B	0.65095 (17)	0.48942 (13)	0.37955 (8)	0.0161 (3)	
C3B	0.67357 (17)	0.53950 (13)	0.32650 (8)	0.0163 (3)	
H3BA	0.6939	0.6126	0.3266	0.020*	
C4B	0.66631 (17)	0.48133 (13)	0.27202 (8)	0.0166 (3)	
C5B	0.64153 (17)	0.37444 (13)	0.27156 (8)	0.0166 (3)	
H5BA	0.6424	0.3354	0.2345	0.020*	
C6B	0.61546 (17)	0.32465 (13)	0.32535 (8)	0.0155 (3)	

### Atomic displacement parameters (Å^2^)

**Table:**

	*U*^11^	*U*^22^	*U*^33^	*U*^12^	*U*^13^	*U*^23^
Cl	0.0243 (2)	0.0218 (2)	0.0298 (2)	−0.00915 (16)	−0.00314 (16)	−0.00647 (16)
S	0.0176 (2)	0.01094 (19)	0.0234 (2)	−0.00117 (14)	0.00049 (15)	0.00361 (14)
O1A	0.0364 (7)	0.0128 (6)	0.0385 (8)	0.0020 (5)	0.0102 (6)	−0.0017 (5)
O2A	0.0292 (7)	0.0219 (6)	0.0323 (7)	−0.0034 (5)	−0.0053 (5)	0.0135 (6)
N1A	0.0132 (6)	0.0127 (6)	0.0238 (7)	0.0000 (5)	0.0027 (5)	0.0008 (5)
N2A	0.0123 (6)	0.0124 (6)	0.0188 (7)	−0.0019 (5)	0.0033 (5)	0.0007 (5)
C1A	0.0130 (7)	0.0158 (8)	0.0221 (8)	−0.0039 (6)	0.0030 (6)	−0.0064 (6)
C2A	0.0162 (8)	0.0114 (7)	0.0291 (9)	−0.0011 (6)	0.0038 (7)	−0.0018 (6)
C3A	0.0117 (7)	0.0131 (7)	0.0168 (7)	−0.0001 (6)	0.0039 (6)	−0.0007 (6)
C4A	0.0125 (7)	0.0129 (7)	0.0175 (7)	−0.0003 (6)	0.0045 (6)	−0.0012 (6)
C5A	0.0145 (7)	0.0163 (8)	0.0172 (8)	0.0005 (6)	0.0018 (6)	−0.0007 (6)
C6A	0.0105 (7)	0.0123 (7)	0.0185 (8)	0.0018 (6)	0.0030 (6)	−0.0020 (6)
C7A	0.0130 (7)	0.0150 (7)	0.0153 (7)	0.0005 (6)	0.0027 (6)	−0.0004 (6)
C8A	0.0117 (7)	0.0152 (7)	0.0180 (7)	0.0033 (6)	0.0017 (6)	−0.0024 (6)
C9A	0.0161 (8)	0.0206 (8)	0.0288 (9)	−0.0015 (7)	−0.0033 (7)	−0.0007 (7)
C10A	0.0148 (7)	0.0176 (8)	0.0167 (7)	0.0023 (6)	0.0019 (6)	0.0025 (6)
C11A	0.0142 (7)	0.0136 (7)	0.0197 (8)	0.0003 (6)	0.0046 (6)	0.0010 (6)
C12A	0.0095 (7)	0.0129 (7)	0.0197 (8)	0.0009 (6)	−0.0001 (6)	−0.0001 (6)
C13A	0.0150 (7)	0.0163 (8)	0.0166 (8)	0.0020 (6)	0.0009 (6)	−0.0022 (6)
C14A	0.0152 (7)	0.0176 (8)	0.0152 (7)	0.0012 (6)	0.0014 (6)	0.0028 (6)
C15A	0.0107 (7)	0.0105 (7)	0.0220 (8)	0.0005 (5)	0.0003 (6)	0.0018 (6)
C16A	0.0167 (7)	0.0133 (7)	0.0166 (7)	0.0015 (6)	0.0019 (6)	−0.0017 (6)
C17A	0.0158 (7)	0.0158 (8)	0.0160 (8)	0.0011 (6)	0.0023 (6)	0.0016 (6)
C18A	0.0179 (8)	0.0285 (10)	0.0304 (10)	−0.0082 (7)	0.0004 (7)	0.0005 (8)
O1B	0.0329 (7)	0.0235 (6)	0.0228 (6)	−0.0118 (5)	0.0096 (5)	0.0004 (5)
O2B	0.0533 (9)	0.0336 (8)	0.0193 (7)	−0.0108 (7)	−0.0028 (6)	0.0006 (6)
O3B	0.0597 (10)	0.0230 (7)	0.0363 (8)	0.0120 (7)	0.0073 (7)	−0.0082 (6)
O4B	0.0237 (6)	0.0217 (6)	0.0238 (6)	−0.0061 (5)	−0.0010 (5)	0.0080 (5)
O5B	0.0535 (9)	0.0311 (8)	0.0200 (7)	−0.0065 (7)	0.0119 (6)	−0.0009 (6)
O6B	0.0330 (7)	0.0204 (6)	0.0288 (7)	−0.0022 (5)	0.0043 (6)	−0.0056 (5)
O7B	0.0353 (7)	0.0176 (6)	0.0341 (7)	−0.0001 (5)	0.0136 (6)	0.0070 (5)
N1B	0.0216 (7)	0.0219 (7)	0.0213 (8)	−0.0024 (6)	0.0052 (6)	−0.0023 (6)
N2B	0.0166 (7)	0.0219 (7)	0.0203 (7)	−0.0028 (6)	0.0004 (5)	0.0040 (6)
N3B	0.0140 (6)	0.0153 (7)	0.0282 (8)	−0.0015 (5)	0.0029 (6)	0.0006 (6)
C1B	0.0102 (7)	0.0176 (8)	0.0204 (8)	−0.0020 (6)	0.0009 (6)	0.0033 (6)
C2B	0.0115 (7)	0.0171 (8)	0.0193 (8)	0.0008 (6)	0.0004 (6)	−0.0018 (6)
C3B	0.0112 (7)	0.0146 (8)	0.0223 (8)	−0.0001 (6)	−0.0001 (6)	0.0023 (6)
C4B	0.0123 (7)	0.0190 (8)	0.0181 (8)	−0.0019 (6)	0.0011 (6)	0.0035 (6)
C5B	0.0117 (7)	0.0190 (8)	0.0186 (8)	−0.0007 (6)	0.0002 (6)	−0.0011 (6)
C6B	0.0108 (7)	0.0134 (7)	0.0219 (8)	−0.0020 (6)	0.0005 (6)	0.0014 (6)

### Geometric parameters (Å, °)

**Table:**

Cl—C1A	1.7363 (16)	C12A—C17A	1.402 (2)
S—O1A	1.4355 (14)	C13A—C14A	1.392 (2)
S—O2A	1.4436 (13)	C13A—H13A	0.9500
S—C18A	1.7640 (19)	C14A—C15A	1.394 (2)
S—C15A	1.7679 (16)	C14A—H14A	0.9500
N1A—C2A	1.333 (2)	C15A—C16A	1.388 (2)
N1A—C3A	1.347 (2)	C16A—C17A	1.392 (2)
N2A—C7A	1.340 (2)	C16A—H16A	0.9500
N2A—C8A	1.348 (2)	C17A—H17A	0.9500
N2A—H2AB	0.8800	C18A—H18A	0.9800
C1A—C5A	1.381 (2)	C18A—H18B	0.9800
C1A—C2A	1.388 (2)	C18A—H18C	0.9800
C2A—H2AA	0.9500	O1B—C1B	1.242 (2)
C3A—C4A	1.405 (2)	O2B—N1B	1.219 (2)
C3A—C6A	1.489 (2)	O3B—N1B	1.224 (2)
C4A—C5A	1.398 (2)	O4B—N2B	1.2305 (19)
C4A—C12A	1.490 (2)	O5B—N2B	1.234 (2)
C5A—H5AA	0.9500	O6B—N3B	1.233 (2)
C6A—C7A	1.386 (2)	O7B—N3B	1.231 (2)
C6A—C11A	1.400 (2)	N1B—C2B	1.461 (2)
C7A—H7AA	0.9500	N2B—C4B	1.441 (2)
C8A—C10A	1.387 (2)	N3B—C6B	1.452 (2)
C8A—C9A	1.490 (2)	C1B—C6B	1.452 (2)
C9A—H9AA	0.9800	C1B—C2B	1.464 (2)
C9A—H9AB	0.9800	C2B—C3B	1.361 (2)
C9A—H9AC	0.9800	C3B—C4B	1.394 (2)
C10A—C11A	1.384 (2)	C3B—H3BA	0.9500
C10A—H10A	0.9500	C4B—C5B	1.381 (2)
C11A—H11A	0.9500	C5B—C6B	1.383 (2)
C12A—C13A	1.392 (2)	C5B—H5BA	0.9500
			
O1A—S—O2A	118.49 (9)	C12A—C13A—C14A	119.75 (15)
O1A—S—C18A	109.69 (9)	C12A—C13A—H13A	120.1
O2A—S—C18A	107.39 (9)	C14A—C13A—H13A	120.1
O1A—S—C15A	109.14 (8)	C13A—C14A—C15A	119.21 (15)
O2A—S—C15A	108.00 (8)	C13A—C14A—H14A	120.4
C18A—S—C15A	103.01 (8)	C15A—C14A—H14A	120.4
C2A—N1A—C3A	119.18 (14)	C16A—C15A—C14A	121.74 (15)
C7A—N2A—C8A	123.92 (14)	C16A—C15A—S	120.60 (12)
C7A—N2A—H2AB	118.0	C14A—C15A—S	117.54 (12)
C8A—N2A—H2AB	118.0	C15A—C16A—C17A	118.62 (15)
C5A—C1A—C2A	120.25 (15)	C15A—C16A—H16A	120.7
C5A—C1A—Cl	120.11 (13)	C17A—C16A—H16A	120.7
C2A—C1A—Cl	119.60 (13)	C16A—C17A—C12A	120.24 (15)
N1A—C2A—C1A	121.42 (15)	C16A—C17A—H17A	119.9
N1A—C2A—H2AA	119.3	C12A—C17A—H17A	119.9
C1A—C2A—H2AA	119.3	S—C18A—H18A	109.5
N1A—C3A—C4A	122.41 (14)	S—C18A—H18B	109.5
N1A—C3A—C6A	114.07 (14)	H18A—C18A—H18B	109.5
C4A—C3A—C6A	123.47 (14)	S—C18A—H18C	109.5
C5A—C4A—C3A	117.60 (14)	H18A—C18A—H18C	109.5
C5A—C4A—C12A	119.48 (14)	H18B—C18A—H18C	109.5
C3A—C4A—C12A	122.92 (14)	O2B—N1B—O3B	123.86 (16)
C1A—C5A—C4A	118.72 (15)	O2B—N1B—C2B	118.13 (15)
C1A—C5A—H5AA	120.6	O3B—N1B—C2B	117.87 (15)
C4A—C5A—H5AA	120.6	O4B—N2B—O5B	123.07 (15)
C7A—C6A—C11A	116.81 (14)	O4B—N2B—C4B	118.76 (15)
C7A—C6A—C3A	121.39 (14)	O5B—N2B—C4B	118.16 (14)
C11A—C6A—C3A	121.65 (14)	O7B—N3B—O6B	122.22 (15)
N2A—C7A—C6A	120.60 (14)	O7B—N3B—C6B	119.13 (14)
N2A—C7A—H7AA	119.7	O6B—N3B—C6B	118.59 (14)
C6A—C7A—H7AA	119.7	O1B—C1B—C6B	126.59 (15)
N2A—C8A—C10A	117.52 (14)	O1B—C1B—C2B	121.90 (15)
N2A—C8A—C9A	117.64 (15)	C6B—C1B—C2B	111.45 (14)
C10A—C8A—C9A	124.83 (15)	C3B—C2B—N1B	116.85 (15)
C8A—C9A—H9AA	109.5	C3B—C2B—C1B	124.75 (15)
C8A—C9A—H9AB	109.5	N1B—C2B—C1B	118.40 (14)
H9AA—C9A—H9AB	109.5	C2B—C3B—C4B	118.66 (15)
C8A—C9A—H9AC	109.5	C2B—C3B—H3BA	120.7
H9AA—C9A—H9AC	109.5	C4B—C3B—H3BA	120.7
H9AB—C9A—H9AC	109.5	C5B—C4B—C3B	121.29 (15)
C11A—C10A—C8A	120.01 (15)	C5B—C4B—N2B	118.90 (15)
C11A—C10A—H10A	120.0	C3B—C4B—N2B	119.69 (15)
C8A—C10A—H10A	120.0	C4B—C5B—C6B	119.59 (16)
C10A—C11A—C6A	120.96 (15)	C4B—C5B—H5BA	120.2
C10A—C11A—H11A	119.5	C6B—C5B—H5BA	120.2
C6A—C11A—H11A	119.5	C5B—C6B—N3B	115.48 (15)
C13A—C12A—C17A	120.20 (15)	C5B—C6B—C1B	123.50 (15)
C13A—C12A—C4A	119.49 (14)	N3B—C6B—C1B	121.01 (14)
C17A—C12A—C4A	120.30 (14)		
			
C3A—N1A—C2A—C1A	1.2 (2)	C18A—S—C15A—C16A	93.67 (14)
C5A—C1A—C2A—N1A	−4.2 (3)	O1A—S—C15A—C14A	161.04 (13)
Cl—C1A—C2A—N1A	178.19 (12)	O2A—S—C15A—C14A	30.96 (15)
C2A—N1A—C3A—C4A	4.7 (2)	C18A—S—C15A—C14A	−82.46 (14)
C2A—N1A—C3A—C6A	−172.79 (14)	C14A—C15A—C16A—C17A	2.8 (2)
N1A—C3A—C4A—C5A	−7.5 (2)	S—C15A—C16A—C17A	−173.15 (12)
C6A—C3A—C4A—C5A	169.75 (14)	C15A—C16A—C17A—C12A	1.3 (2)
N1A—C3A—C4A—C12A	171.79 (15)	C13A—C12A—C17A—C16A	−4.9 (2)
C6A—C3A—C4A—C12A	−10.9 (2)	C4A—C12A—C17A—C16A	174.19 (14)
C2A—C1A—C5A—C4A	1.2 (2)	O2B—N1B—C2B—C3B	−146.94 (17)
Cl—C1A—C5A—C4A	178.77 (12)	O3B—N1B—C2B—C3B	28.9 (2)
C3A—C4A—C5A—C1A	4.4 (2)	O2B—N1B—C2B—C1B	32.3 (2)
C12A—C4A—C5A—C1A	−174.96 (14)	O3B—N1B—C2B—C1B	−151.84 (16)
N1A—C3A—C6A—C7A	143.34 (15)	O1B—C1B—C2B—C3B	−167.28 (16)
C4A—C3A—C6A—C7A	−34.2 (2)	C6B—C1B—C2B—C3B	10.0 (2)
N1A—C3A—C6A—C11A	−32.2 (2)	O1B—C1B—C2B—N1B	13.6 (2)
C4A—C3A—C6A—C11A	150.31 (15)	C6B—C1B—C2B—N1B	−169.16 (14)
C8A—N2A—C7A—C6A	0.2 (2)	N1B—C2B—C3B—C4B	173.89 (14)
C11A—C6A—C7A—N2A	−3.6 (2)	C1B—C2B—C3B—C4B	−5.3 (2)
C3A—C6A—C7A—N2A	−179.32 (14)	C2B—C3B—C4B—C5B	−2.1 (2)
C7A—N2A—C8A—C10A	3.2 (2)	C2B—C3B—C4B—N2B	−178.17 (14)
C7A—N2A—C8A—C9A	−177.65 (15)	O4B—N2B—C4B—C5B	179.15 (15)
N2A—C8A—C10A—C11A	−3.0 (2)	O5B—N2B—C4B—C5B	−0.7 (2)
C9A—C8A—C10A—C11A	177.88 (16)	O4B—N2B—C4B—C3B	−4.6 (2)
C8A—C10A—C11A—C6A	−0.4 (2)	O5B—N2B—C4B—C3B	175.46 (15)
C7A—C6A—C11A—C10A	3.6 (2)	C3B—C4B—C5B—C6B	3.5 (2)
C3A—C6A—C11A—C10A	179.36 (14)	N2B—C4B—C5B—C6B	179.62 (14)
C5A—C4A—C12A—C13A	−53.2 (2)	C4B—C5B—C6B—N3B	−176.48 (14)
C3A—C4A—C12A—C13A	127.46 (17)	C4B—C5B—C6B—C1B	2.3 (2)
C5A—C4A—C12A—C17A	127.70 (16)	O7B—N3B—C6B—C5B	173.90 (14)
C3A—C4A—C12A—C17A	−51.6 (2)	O6B—N3B—C6B—C5B	−3.5 (2)
C17A—C12A—C13A—C14A	4.4 (2)	O7B—N3B—C6B—C1B	−4.9 (2)
C4A—C12A—C13A—C14A	−174.62 (14)	O6B—N3B—C6B—C1B	177.70 (14)
C12A—C13A—C14A—C15A	−0.5 (2)	O1B—C1B—C6B—C5B	168.74 (16)
C13A—C14A—C15A—C16A	−3.2 (2)	C2B—C1B—C6B—C5B	−8.4 (2)
C13A—C14A—C15A—S	172.85 (12)	O1B—C1B—C6B—N3B	−12.5 (3)
O1A—S—C15A—C16A	−22.84 (15)	C2B—C1B—C6B—N3B	170.35 (13)
O2A—S—C15A—C16A	−152.91 (13)		

### Hydrogen-bond geometry (Å, °)

**Table:**

*D*—H···*A*	*D*—H	H···*A*	*D*···*A*	*D*—H···*A*
N2A—H2AB···O1B	0.88	1.79	2.6588 (18)	172.
N2A—H2AB···O7B	0.88	2.46	2.8898 (19)	111.
C2A—H2AA···O1A^i^	0.95	2.56	3.455 (2)	156.
C9A—H9AA···O1B	0.98	2.60	3.357 (2)	134.
C13A—H13A···O2A^ii^	0.95	2.35	3.294 (2)	173.
C18A—H18C···O2B^iii^	0.98	2.38	3.249 (2)	147.
C5A—H5AA···O6B^iv^	0.95	2.45	3.329 (2)	153.
C7A—H7AA···O4B^v^	0.95	2.52	3.326 (2)	143.

Symmetry codes: (i) *x*, *y*−1, *z*; (ii) −*x*, *y*−1/2, −*z*+1/2; (iii) −*x*+1, −*y*+1, −*z*+1; (iv) *x*−1, *y*, *z*; (v) −*x*+1, *y*−1/2, −*z*+1/2.

### Table 2 Cg···Cg π-stacking interactions, Cg2, Cg3 and Cg4 are the centroids of rings N2A/C7A/C6A/C11A/C10A/C8A, C12A—C17A and C1B—C6B [Symmetry codes: (i) 1-x, -y, 1-z; (ii) -1+x, y, z]

**Table:**

*Cg*I···*Cg*J	Cg···Cg (Å)	CgI Perp (Å)	CgJ Perp (Å)	Slippage (Å)
Cg2···Cg2^i^	3.8192 (9)	-3.4367 (6)	-3.4367 (6)	1.66 (6)
Cg3···Cg4^ii^	3.6749 (9)	3.5169 (7)	-3.3977 (6)	
